# Crystalloids versus colloids for goal-directed fluid therapy in major surgery

**DOI:** 10.1186/cc7761

**Published:** 2009-03-21

**Authors:** Luzius B Hiltebrand, Oliver Kimberger, Michael Arnberger, Sebastian Brandt, Andrea Kurz, Gisli H Sigurdsson

**Affiliations:** 1 Department of Anaesthesiology and Pain Therapy, Inselspital, Bern University Hospital, Freiburgstrasse, Bern, CH 3010, Switzerland; 2 Department of Anaesthesia, General Intensive Care and Pain Medicine, Medical University of Vienna, Währinger Gürtel 18-20, Vienna, A 1090, Austria; 3 Department of Outcomes Research, The Cleveland Clinic, 9500 Euclid Avenue, Cleveland, OH 44195, USA; 4 Department of Anaesthesia and Intensive Care Medicine, Landspitali University Hospital, and University of Iceland, Hringbraut, Reykjavik, IS 101, Iceland

## Abstract

**Introduction:**

Perioperative hypovolemia arises frequently and contributes to intestinal hypoperfusion and subsequent postoperative complications. Goal-directed fluid therapy might reduce these complications. The aim of this study was to compare the effects of goal-directed administration of crystalloids and colloids on the distribution of systemic, hepatosplanchnic, and microcirculatory (small intestine) blood flow after major abdominal surgery in a clinically relevant pig model.

**Methods:**

Twenty-seven pigs were anesthetized and mechanically ventilated and underwent open laparotomy. They were randomly assigned to one of three treatment groups: the restricted Ringer lactate (R-RL) group (n = 9) received 3 mL/kg per hour of RL, the goal-directed RL (GD-RL) group (n = 9) received 3 mL/kg per hour of RL and intermittent boluses of 250 mL of RL, and the goal-directed colloid (GD-C) group (n = 9) received 3 mL/kg per hour of RL and boluses of 250 mL of 6% hydroxyethyl starch (130/0.4). The latter two groups received a bolus infusion when mixed venous oxygen saturation was below 60% ('lockout' time of 30 minutes). Regional blood flow was measured in the superior mesenteric artery and the celiac trunk. In the small bowel, microcirculatory blood flow was measured using laser Doppler flowmetry. Intestinal tissue oxygen tension was measured with intramural Clark-type electrodes.

**Results:**

After 4 hours of treatment, arterial blood pressure, cardiac output, mesenteric artery flow, and mixed oxygen saturation were significantly higher in the GD-C and GD-RL groups than in the R-RL group. Microcirculatory flow in the intestinal mucosa increased by 50% in the GD-C group but remained unchanged in the other two groups. Likewise, tissue oxygen tension in the intestine increased by 30% in the GD-C group but remained unchanged in the GD-RL group and decreased by 18% in the R-RL group. Mesenteric venous glucose concentrations were higher and lactate levels were lower in the GD-C group compared with the two crystalloid groups.

**Conclusions:**

Goal-directed colloid administration markedly increased microcirculatory blood flow in the small intestine and intestinal tissue oxygen tension after abdominal surgery. In contrast, goal-directed crystalloid and restricted crystalloid administrations had no such effects. Additionally, mesenteric venous glucose and lactate concentrations suggest that intestinal cellular substrate levels were higher in the colloid-treated than in the crystalloid-treated animals. These results support the notion that perioperative goal-directed therapy with colloids might be beneficial during major abdominal surgery.

## Introduction

Perioperative care of high-risk surgical patients remains a challenge. Despite improvements in perioperative management, the rate of severe complications after major surgery remains high [1,2]. It has been shown that perioperative decrease in oxygen transport is closely related to the development of organ failure and death [3,4]. Failure of adequate fluid therapy is a common cause of decreased oxygen transport [3,5,6]. Intraoperative gut hypoperfusion was identified in 63% of major surgery patients and was associated with increased morbidity and hospital stay [3]. As a consequence, low gastric intramucosal pH assessed by gastric tonometry was among the strongest predictors of various perioperative complications [3,7].

Although the importance of normovolemia is widely accepted, there is an ongoing debate about the right amount and the right type of fluid to be administered perioperatively in major surgery. Several recent publications have suggested that goal-directed fluid therapy [8-10] with crystalloid or colloid administration is a possible way to decrease morbidity and mortality in major surgery patients. Despite reports of decreased morbidity and mortality [5,8,11,12] in these studies, the actual effect of a perioperative goal-directed fluid therapy and, in particular, effects of the kind of fluid (namely, crystalloid or colloid solution) on the small bowel – the motor of multiorgan failure – are still largely unknown. Goal-directed fluid therapy with colloids has been shown to improve gastric tonometry values in patients after cardiac surgery, suggesting improved gastric perfusion [5]. On the other hand, distribution of blood flow after a fluid challenge is heterogeneous and increased cardiac output does not automatically result in increased hepatosplanchnic blood flow [13]. Thus, the question of which way perioperative goal-directed fluid therapy influences regional and microcirculatory blood flow as well as tissue oxygen tension in the gastrointestinal tract remains unresolved. Additionally, the type of fluid administered is likely to play an important role [14].

In the present study, we hypothesize that goal-directed colloid fluid therapy in the setting of major abdominal surgery increases intestinal microcirculatory blood flow and tissue oxygen tension. The main aim of this study was to investigate the influence of three different fluid management strategies on systemic blood flow (cardiac index, or CI), regional blood flow (hepatosplanchnic flow), local blood flow (microcirculatory flow in the small intestine), and intestinal tissue oxygen tension in a pig model of major abdominal surgery. An additional aim was to identify possible differences in effects between crystalloid- and colloid-based fluid treatments.

## Materials and methods

This study was performed in accordance with the National Institutes of Health (Bethesda, MD, USA) guidelines for the care and use of experimental animals. The protocol was approved by the animal ethics committee of Canton Bern, Switzerland. Twenty-seven domestic pigs (weight 28 to 32 kg) were fasted overnight but had free access to water. The pigs were sedated with intramuscular ketamine (20 mg/kg) and xylazine (2 mg/kg). Then a peripheral intravenous catheter was inserted in an ear vein for initial administration of fluids and medications. Anesthesia was induced with midazolam 0.4 mg/kg and atropine 1 mg. After induction, the pigs were orally intubated and ventilated with oxygen in air (fraction of inspired oxygen = 0.3). Anesthesia was maintained with midazolam 0.5 mg/kg per hour, fentanyl 15 μg/kg per hour, pancuronium 0.3 mg/kg per hour, and low-dose propofol 0.15 mg/kg per hour. The animals were ventilated with a volume-controlled ventilator with a positive end-expiratory pressure of 5 cm H_2_O (Servo 900C; Siemens, Solna, Sweden). Tidal volume was kept at 8 to 10 mL/kg, and the respiratory rate was adjusted (22 to 26 breaths per minute) to maintain end-tidal carbon dioxide tension (PaCO_2_) at 5.3 ± 0.5 kPa. Immediately after induction, all animals received 1.5 g of Cefuroxim intravenously as an antibiotic prophylaxis. The stomach was emptied with a large-bore orogastric tube.

### Surgical preparation

Through a left cervical cut-down, indwelling catheters were inserted into the left carotid artery and superior vena cava. A balloon-tipped catheter was inserted into the pulmonary artery through the right external jugular vein. Location of the catheter tip was determined by observing the characteristic pressure trace on the monitor as the catheter was advanced through the right heart into the pulmonary artery. Similarly, a fiberoptic hepatic vein catheter was inserted through the right jugular vein. Correct positioning was verified by a 15% to 20% decrease in the continuously measured hepatic vein saturation versus the mixed venous saturation and by a significant decrease in lactate concentration compared with mixed venous blood. The right carotid artery was dissected free and a 4-mm ultrasound transit time flow probe was placed around the vessel to measure carotid artery blood flow.

With the pig in the supine position, a midline laparotomy was performed. A catheter was inserted into the urinary bladder for drainage of urine. A second catheter was inserted into the mesenteric vein for blood sampling. The superior mesenteric artery (SMA), the celiac trunk, and the hepatic artery were identified close to their origin. After dissection to free these vessels from the surrounding tissues, precalibrated ultrasonic transit time flow probes (Transonic Systems, Ithaca, NY, USA) were placed around the vessels and connected to an ultrasound blood flowmeter (T 207; Transonic Systems).

Through a small incision in the jejunum, a custom-made laser Doppler flowmetry (LDF) probe (Oxford Optronix, Oxford, UK) was sutured to the jejunum mucosa for measurements of microcirculatory blood flow in the mucosa. A second LDF probe was sutured to the adjacent jejunum muscularis. Both LDF probes were attached with six microsutures to ensure continuous and steady contact with the tissue under investigation, preventing motion disturbance from respiration and gastrointestinal movements throughout the experiment. The signals of the LDF probes were visualized on a computer monitor. If the signal quality of a probe was poor, the position of the probe was corrected immediately. The incision in the jejunum also allowed controlled positioning of an air tonometer tube (TRIP Sigmoid catheter; Datex-Ohmeda, GE Healthcare, Helsinki, Finland). The bowel incision was then closed with continuous sutures.

For intramural intestinal tissue oxygen tension measurement, a polarographic tissue oxygen tension sensor was inserted into a section of healthy jejunum between the serosal and the mucosal tissue planes. The method has been described previously [15,16]. Care was taken to minimize handling of the small intestine and to return the bowel to a neutral position. After preparation, the abdominal incision was closed and the animals were allowed to recover from instrumentation and stabilize for 60 minutes.

Throughout the entire study, all animals received a basal infusion of 3 mL/kg per hour of Ringer lactate (RL) to avoid excessive fluid administration. This fixed fluid administration resulted in a low central venous and pulmonary capillary wedge pressure (PCWP) of between 2 and 4 mm Hg at baseline. Body temperature of the animals was maintained at 38.0 ± 0.5°C with a forced-air patient air warming system (Warm Touch 5700; Mallinckrodt, Hennef, Germany). Baseline measurements were performed after stabilization at t = 0 minutes. Subsequently, all hemodynamic measurements were repeated every 30 minutes for 4 hours. Blood samples were drawn hourly after the measurements of the hemodynamic parameters.

Immediately after baseline measurements, the pigs were randomly assigned to one of three fluid treatment groups using a reproducible set of computer-generated random numbers. The assignments were kept in sealed, opaque, and sequentially numbered envelopes until used. Once the fluid therapy was assigned, the investigators were not blinded anymore. The assigned fluid therapy was started 15 minutes after the first measurement. The fluid treatment groups were as follows.

### Groups

The 'restricted Ringer lactate' (R-RL) group (n = 9) received a fixed administration of 3 mL/kg per hour of lactated Ringer solution throughout the experiment without additional fluids.

The 'goal-directed Ringer lactate' (GD-RL) group (n = 9) received a fixed administration of 3 mL/kg per hour of lactated Ringer solution throughout the experiment. Additionally, this group received an administration of 250 mL of lactated Ringer solution as a bolus (within 3 to 4 minutes) if the mixed venous oxygen saturation (SvO_2_) was less than 60% ('lockout' time between two boluses = 30 minutes).

The 'goal-directed colloid' (GD-C) group (n = 9) received a fixed administration of 3 mL/kg per hour of lactated Ringer solution throughout the experiment. Additionally, this group received an administration of 250 mL of hydroxyethyl starch (HES) (130/0.4) as a bolus (within 3 to 4 minutes) if the SvO_2 _was less than 60% (lockout time between two boluses = 30 minutes).

### Measurements

#### Respiratory monitoring

Expired minute volume, tidal volume, respiratory rate, peak and other respiratory pressures, positive end-expiratory pressure, inspired and end-tidal carbon dioxide fraction, and inspired/expired oxygen fraction were monitored (S/5 Critical Care Monitor; Datex-Ohmeda, GE Healthcare) throughout the study.

#### Hemodynamic monitoring

Mean arterial blood pressure (MAP) (mm Hg), central venous pressure (CVP) (mm Hg), mean pulmonary artery pressure (PAP) (mm Hg), hepatic vein pressure (HVP) (mm Hg), and PCWP (mm Hg) were recorded with quartz pressure transducers. Pulse pressure variation (PPV) and stroke volume (SV) were measured with a PiCCO (pulse contour cardiac output) plus hemodynamic monitor (Pulsion Medical Systems GmbH, Munich, Germany) connected to the arterial pressure transducer. Heart rate was measured from the electrocardiogram. Heart rate, MAP, PAP, and CVP were displayed continuously on a multi-modular monitor (S/5 Critical Care Monitor). A thermodilution method was used to measure cardiac output at each measurement point (mean value of three consecutive manually performed measurements with 5 mL of cold saline). Core temperature was measured from the thermistor in the pulmonary artery catheter. Regional blood flow in the SMA, the celiac trunk, and the hepatic artery was continuously measured throughout the experiments with ultrasonic transit time flowmetry (mL per minute) using two double-channel HT 206 flowmeters (Transonic Systems).

Microcirculatory blood flow was monitored continuously in the mucosa and the muscularis of the jejunum using a multi-channel laser Doppler flowmeter system (Oxford Optronix). A detailed description of the theory of LDF operation and practical details of LDF measurements have been published previously [17,18]. The regional blood flow and the LDF data were acquired online with a sampling rate of 10 Hz via a multi-channel interface (MP 150; Biopac Systems Inc., Goleta, CA, USA) with acquisition software (Acqknowledge 3.9; Biopac Systems Inc.) and saved on a portable computer. Laser Doppler flowmeters are not calibrated to measure absolute blood flow but indicate microcirculatory blood flow in arbitrary perfusion units. Due to a relatively large variability of baseline values, the results usually are expressed as changes relative to baseline [19-22] and that was also the case in the present study.

The jejunal intramucosal carbon dioxide pressure was measured with air tonometry (Tonocap^® ^Monitor; Datex-Ohmeda, GE Healthcare). The jejunal mucosal-to-arterial carbon dioxide pressure gap (CO_2 _gap) was calculated at each measurement point.

Arterial, mixed venous, mesenteric, and hepatic venous blood samples were withdrawn hourly from the indwelling catheters and immediately analyzed in a blood gas analyzer (ABL 620; Radiometer, Copenhagen, Denmark) for oxygen partial pressure (pO_2_) (kPa), carbon dioxide partial pressure (pCO_2_) (kPa), pH, lactate (mmol/L), and base excess (BE). Arterial oxygen saturation (SO_2_) (percentage) and total hemoglobin concentration (Hb) (g/dL) were measured with an analyzer specially adjusted to porcine blood (OSM 3; Radiometer). All values were adjusted to body temperature. Mixed and hepatic venous saturations were displayed continuously on two continuous cardiac output monitors (Vigilance; Edwards Lifesciences LLC, Baxter, Irvine, CA, USA).

CI (mL/kg per minute), SMA flow index (SMAI) (mL/kg per minute), and systemic vascular resistance index (SVRI) (mm Hg/kg per minute) were indexed to body weight. SVRI was calculated as: SVRI = (MAP - CVP)/CI [20,23].

Systemic oxygen delivery index (sDO_2_I) (mL/kg per minute), systemic oxygen consumption index (sVO_2_I) (mL/kg per minute), and the corresponding mesenteric (splanchnic) variables (mDO_2_I and mVO_2_I) (mL/kg per minute) were calculated using the following formulas: Systemic (total body) oxygen delivery index (sDO_2_) = (CI × CaO_2_), where CaO_2 _is the arterial oxygen content. Systemic (total body) oxygen consumption index (sVO_2_) = (CI × [CaO_2 _- CvO_2_]), where CvO_2 _is the mixed venous oxygen content. Mesenteric (splanchnic) oxygen delivery index (mDO_2_) = SMAI × CaO_2_. Mesenteric (splanchnic) oxygen consumption index (mVO_2_) = SMAI × (CaO_2 _- CmO_2_), where CmO_2 _is the mesenteric vein oxygen content. Oxygen content (mL of O_2_/mL of blood) = ([pO_2 _× 0.0031] + [Hb × SO_2 _× 1.36])/100.

In the same animals an additional hypothesis was tested regarding the changes of microcirculatory blood flow in healthy colon and in a critically perfused colon anastomosis. This data is published elsewhere [24].

### Statistical analysis

Data were tested for normality by QQ-plot and Kolmogorov-Smirnov test. All baseline data (that is, before the start of the respective treatment at t = 0 minutes) were compared with analysis of variance (ANOVA) or Kruskal-Wallis test to exclude initial group discrepancies. Differences between the three fluid treatment groups were assessed by ANOVA for repeated measurements using group as between-subject factor and time as within-subject factor. If a significant difference between the groups was detected, a Tukey *post hoc *test was performed to assess differences at individual time points. Additionally, the area under the variable-time curve for each variable of interest was calculated and compared with ANOVA for group differences. A Tukey *post hoc *test was performed to compare individual treatments if the ANOVA had detected significant differences between the groups. Measurements of microcirculatory blood flow (LDF) were transformed with baseline set to 100% (t = 0 minutes) prior to statistical analysis. Absolute values were used for all other calculations. Data are presented as means ± standard deviations unless otherwise specified. A *P *value of less than 0.05 was considered significant. For statistical calculations, SAS version 8 (SAS Institute Inc., Cary, NC, USA) was used.

## Results

All animals survived until the end of the experiment and were included in the final data analysis. The continuous intravenous infusions of basal RL administered during the entire experiments (induction until the end of the study) to the R-RL, GD-RL, and GD-C groups were 924 ± 44, 943 ± 68, and 917 ± 41 mL, respectively. RL administered as repeated bolus infusions (triggered by an SvO_2 _of less than 60%) was 1,794 ± 211 mL in the GD-RL group while the GD-C group received a total of 831 ± 267 mL of 6% HES (130/0.4) as bolus infusions.

**Figure 1:**
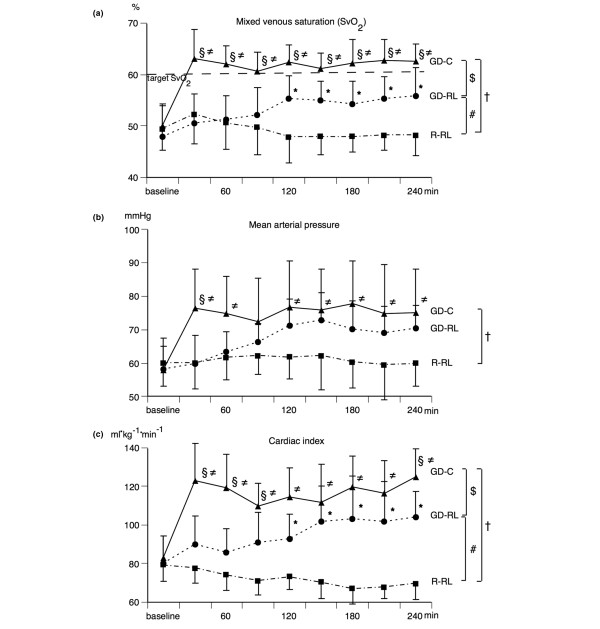
Systemic hemodynamic parameters. **(a) **Changes in mixed venous oxygen saturation (SvO_2_) (mean ± SD) before (baseline) and during the different fluid treatment strategies. SvO_2 _was the target parameter for fluid administration. **(b) **Changes in mean arterial pressure (mean ± SD) before (baseline) and during the different fluid treatment strategies. **(c) **Changes in cardiac index (mean ± SD) before (baseline) and during the different fluid treatment strategies. The restricted Ringer lactate fluid therapy (R-RL) group received 3 mL/kg per hour of lactated Ringer solution throughout the entire experiment. The goal-directed Ringer lactate fluid therapy (GD-RL) group received 3 mL/kg per hour of lactated Ringer solution plus 250 mL of lactated Ringer solution if SvO_2 _was less than 60%. The goal-directed colloid fluid therapy (GD-C) group received 3 mL/kg per hour of lactated Ringer solution plus 250 mL of hydroxyethyl starch (130/0.4) if SvO_2 _was less than 60%. Significant differences (*P *< 0.05) for area under the curve: ^#^R-RL versus GD-RL, ^†^R-RL versus GD-C, ^$^GD-RL versus GD-C. Significant differences (*P *< 0.05) for analysis of variance for repeated measurements (Tukey *post hoc *test): *R-RL versus GD-RL, ^≠^R-RL versus GD-C, ^§^GD-RL versus GD-C. SD, standard deviation.

Systemic hemodynamic data are presented in Figure 1 and Table 1. At baseline, there were no significant differences between the three groups in any parameter measured. In the R-RL group, SvO_2 _was 49.5 ± 4.0% at baseline and remained low (Figure 1). The target value of 60% was not reached in any of the animals in this group at any time point. In the GD-RL group, SvO_2 _increased over time and was 56 ± 5% after 4 hours. Only in three out of nine animals was the target value reached in this group. In the GD-C group, SvO_2 _increased to 63 ± 4% after the first bolus and remained high. The target value for SvO_2 _was reached in all nine animals in this group.

**Table 1:** Systemic, regional, and local hemodynamic variables

	Heart rate^a, b ^(beats per minute)	SV^b ^(mL/beat)	SVRI^a, b ^(mm Hg/kg per minute)	CVP (mm Hg)	HVP (mm Hg)	PCWP^b ^(mm Hg)	CeliacusI (mL/kg per minute)	MBF JM^a, b ^(percentage of baseline)
Restricted Ringer lactate solution (R-RL)								
0 minutes	117 ± 2	28.1 ± 8.4	732 ± 84	2.8 ± 1	3.8 ± 1.4	3.1 ± 0.6	4.0 ± 0.9	100 ± 0
30 minutes	117 ± 4	26.6 ± 6.7	744 ± 123	3.1 ± 0.8	4.5 ± 1	3.3 ± 0.7	4.1 ± 1.0	93 ± 20
180 minutes	123 ± 15	23.7 ± 5.7	868 ± 161	3.3 ± 0.7	3.9 ± 1.4	3.2 ± 0.9	4.9 ± 1.2	74 ± 24
240 minutes	128 ± 14	24.2 ± 5.2	835 ± 149	2.8 ± 1.1	3.9 ± 0.9	2.9 ± 0.7	5.1 ± 1.1	71 ± 18

Goal-directed Ringer lactate solution (GD-RL)								
0 minutes	110 ± 11	25.4 ± 6.6	705 ± 140	3 ± 1.1	4.3 ± 1.4	3.3 ± 1.1	3.8 ± 1.4	100 ± 0
30 minutes	101 ± 4	28.3 ± 7	652 ± 157	3.3 ± 1.1	4.6 ± 1.1	3.6 ± 1	3.9 ± 1.5	97 ± 22
180 minutes	106 ± 15^a^	30.8 ± 6.5	666 ± 147	3.8 ± 1.1	5.5 ± 0.9	3.9 ± 1.2	6.2 ± 1.7	54 ± 18
240 minutes	103 ± 18^a, c^	33.2 ± 6.7	646 ± 90^a^	4 ± 0.9	5.6 ± 1	4.4 ± 1.2	5.8 ± 1.1	49 ± 11

Goal-directed colloid solution (GD-C)								
0 minutes	113 ± 7	25.2 ± 9.8	682 ± 155	3 ± 0.7	4.1 ± 0.9	3.3 ± 0.5	4.3 ± 1.3	100 ± 0
30 minutes	98 ± 9	38.7 ± 7.3^d^	589 ± 76	4.3 ± 0.7^d^	5.4 ± 1	4.6 ± 0.8	5.0 ± 1.5	122 ± 19
180 minutes	106 ± 16^d^	35.1 ± 11^d^	622 ± 109^d^	3.8 ± 1.1	5.1 ± 1	3.9 ± 1.1	5.4 ± 1.5	101 ± 19^d, e^
240 minutes	109 ± 20^d^	33.9 ± 12	563 ± 53^d^	4.2 ± 0.9^d^	5.7 ± 0.9	3.7 ± 0.9	5.4 ± 1.5	94 ± 22^d, e^

Data are presented as mean ± standard deviation. Microcirculatory blood flow was set at 100% at t = 0 minutes. t = 0 baseline values are from before the start of the respective fluid therapy. At t = 30 minutes, effects of one fluid bolus, 250 mL of lactated Ringer solution in the GD-LR group or hydroxyethyl starch in the GD-C group, are presented. At t = 240 minutes, effects after an additional 1,794 ± 211 mL of lactated Ringer solution in the GD-LR group and an additional 831 ± 267 mL of hydroxyethyl starch (130/0.4) in the GD-C group are presented. Significant differences (*P *< 0.05) for area under the curve: ^a^R-RL versus GD-RL, ^b^R-RL versus GD-C. Significant differences (*P *< 0.05) for analysis of variance for repeated measurements (Tukey *post hoc *test): ^c^R-RL versus GD-RL, ^d^R-RL versus GD-C, ^e^GD-RL versus GD-C. The R-RL group received 3 mL/kg per hour of lactated Ringer solution throughout the entire experiment. The GD-RL group received 3 mL/kg per hour of lactated Ringer solution plus 250 mL of lactated Ringer solution if SvO_2 _was less than 60%. The GD-C group received 3 mL/kg per hour of lactated Ringer solution plus 250 mL of hydroxyethyl starch (130/0.4) if SvO_2 _was less than 60%. CeliacusI, truncus celiacus flow index; CVP, central venous pressure; HVP, hepatic venous pressure; MBF JM, microcirculatory blood flow in the muscularis of the jejunum; PCWP, pulmonary capillary wedge pressure; SV, stroke volume; SVRI, systemic vascular resistance index.

In the R-RL group, CI, SV, PPV, MAP, PAP, CVP, hepatic venous pressure (HVP), and PCWP remained largely unchanged. In the GD-RL group, CI and MAP increased slowly (by 15%) over the 4 hours of observation time. SV increased continuously (by greater than 30%) during the study. In the GD-C group, CI and MAP increased by 30% already after the first fluid bolus and remained significantly higher than in the GD-RL group. SV increased by more than 50% after the first fluid bolus and decreased slightly thereafter, resulting in almost identical SV compared with the GD-RL group at the end of the study. PPV in the GD-C group decreased sharply after the first bolus, followed by an increase after 60 minutes. During the remainder of the study, PPV values in the two goal-directed groups were similar and decreased over time. Filling pressures (that is, PAP, CVP, HVP, and PCWP) increased similarly in the GD-RL and GD-C groups.

**Figure 2:**
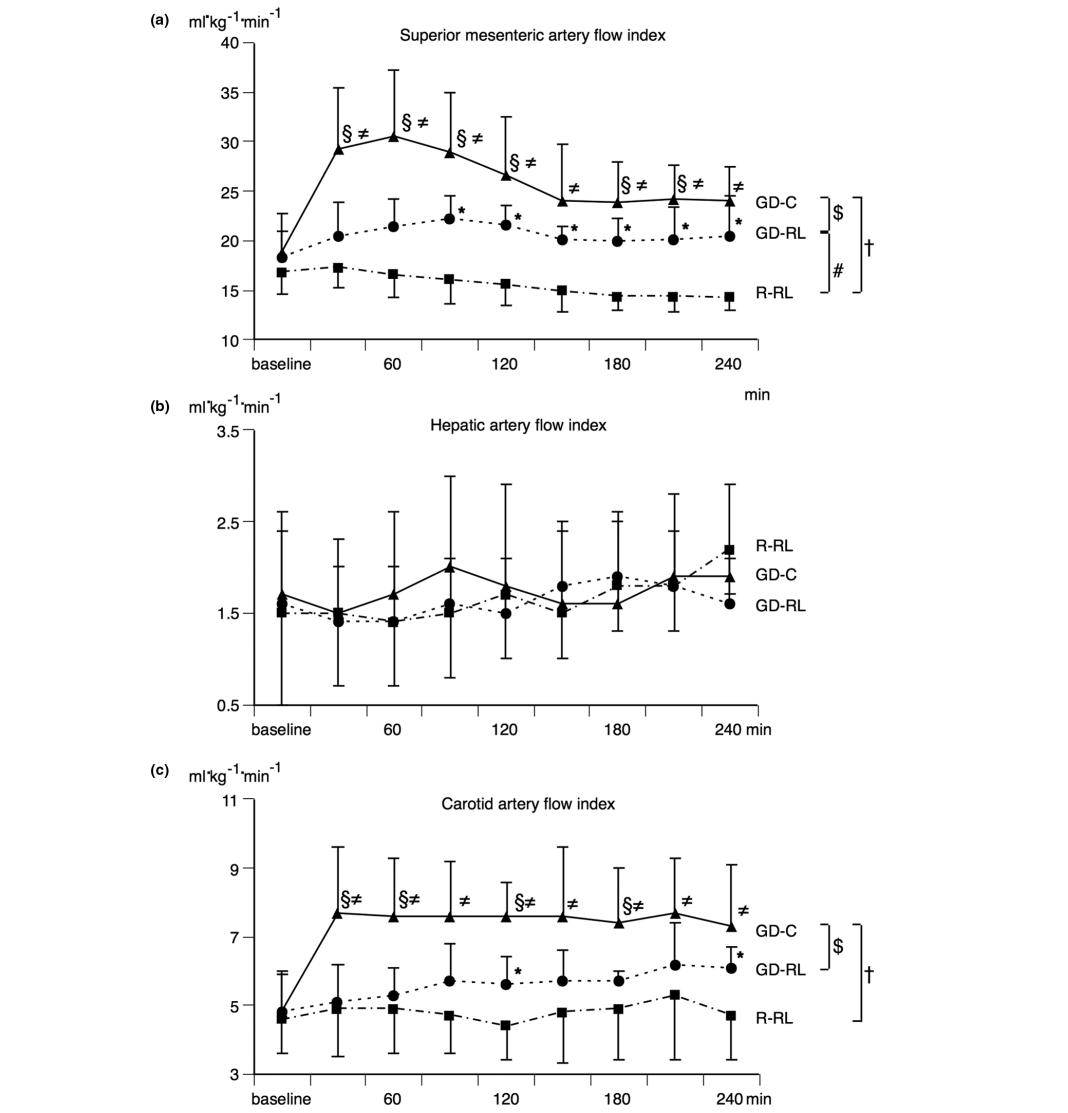
Regional blood flow parameters. **(a) **Changes in superior mesenteric artery flow index (mean ± SD) before (baseline) and during the different fluid treatment strategies. **(b) **Changes in hepatic artery flow index (mean ± SD) before (baseline) and during the different fluid treatment strategies. **(c) **Changes in carotid artery flow index (mean ± SD) before (baseline) and during the different fluid treatment strategies. The restricted Ringer lactate fluid therapy (R-RL) group received 3 mL/kg per hour of lactated Ringer solution throughout the entire experiment. The goal-directed Ringer lactate fluid therapy (GD-RL) group received 3 mL/kg per hour of lactated Ringer solution plus 250 mL of lactated Ringer solution if mixed venous oxygen saturation (SvO_2_) was less than 60%. The goal-directed colloid fluid therapy (GD-C) group received 3 mL/kg per hour of lactated Ringer solution plus 250 mL of hydroxyethyl starch (130/0.4) if SvO_2 _was less than 60%. Significant differences (*P *< 0.05) for area under the curve: ^#^R-RL versus GD-RL, ^†^R-RL versus GD-C, ^$^GD-RL versus GD-C. Significant differences (*P *< 0.05) for analysis of variance for repeated measurements (Tukey *post hoc *test): *R-RL versus GD-RL, ^≠^R-RL versus GD-C, ^§^GD-RL versus GD-C. SD, standard deviation.

Regional blood flow (Figure 2 and Table 1) in the carotid artery was unchanged in the R-RL group but increased by 20% in the GD-RL group and by almost 50% in the GD-C group. On the other hand, blood flow in the celiac trunk and the hepatic artery remained virtually unchanged in all three groups throughout the experiment. SMA flow decreased by 20% in the R-RL group over time but remained nearly unchanged in the GD-RL group. On the other hand, SMAI flow increased significantly in the GD-C group (by 20%).

**Figure 3:**
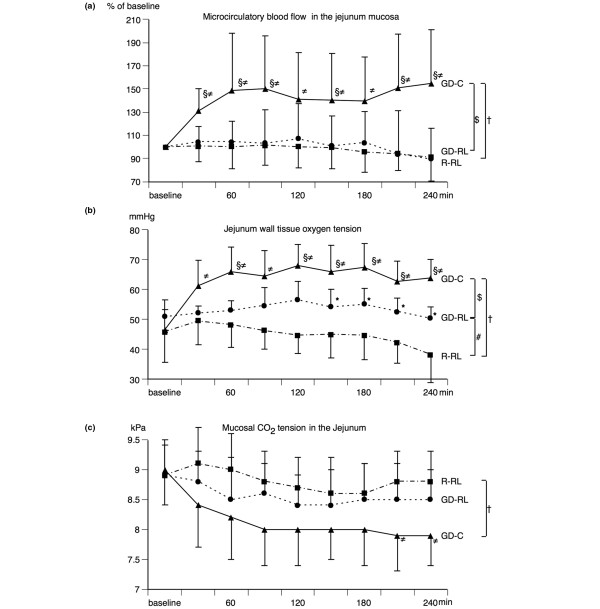
Intestinal perfusion and oxygenation parameters. **(a) **Relative changes in microcirculatory blood flow in the jejunum mucosa (mean ± SD) before (baseline) and during the different fluid treatment strategies. Blood flow was set at 100% at baseline. **(b) **Changes in jejunum wall tissue oxygen tension (mean ± SD) before (baseline) and during the different fluid treatment strategies. **(c) **Changes in mucosal carbon dioxide tension in the jejunum (mean ± SD) before (baseline) and during the different fluid treatment strategies. The restricted Ringer lactate fluid therapy (R-RL) group received 3 mL/kg per hour of lactated Ringer solution throughout the entire experiment. The goal-directed Ringer lactate fluid therapy (GD-RL) group received 3 mL/kg per hour of lactated Ringer solution plus 250 mL of lactated Ringer solution if mixed venous oxygen saturation (SvO_2_) was less than 60%. The goal-directed colloid fluid therapy (GD-C) group received 3 mL/kg per hour of lactated Ringer solution plus 250 mL of hydroxyethyl starch (130/0.4) if SvO_2 _was less than 60%. Significant differences (*P *< 0.05) for area under the curve: ^#^R-RL versus GD-RL, ^†^R-RL versus GD-C, ^$^GD-RL versus GD-C. Significant differences (*P *< 0.05) for analysis of variance for repeated measurements (Tukey *post hoc *test): *R-RL versus GD-RL, ^≠^R-RL versus GD-C, ^§^GD-RL versus GD-C. SD, standard deviation.

Microcirculatory blood flow in the jejunum mucosa (Figure 3) remained largely unchanged in the R-RL and GD-RL groups throughout the 4 hours of treatment but rapidly increased by up to 50% in the GD-C group and remained high until the end of the experiments. Microcirculatory blood flow in the jejunum muscularis (Table 1) remained unchanged in the GD-C group but decreased significantly in the other two groups.

**Figure 4:**
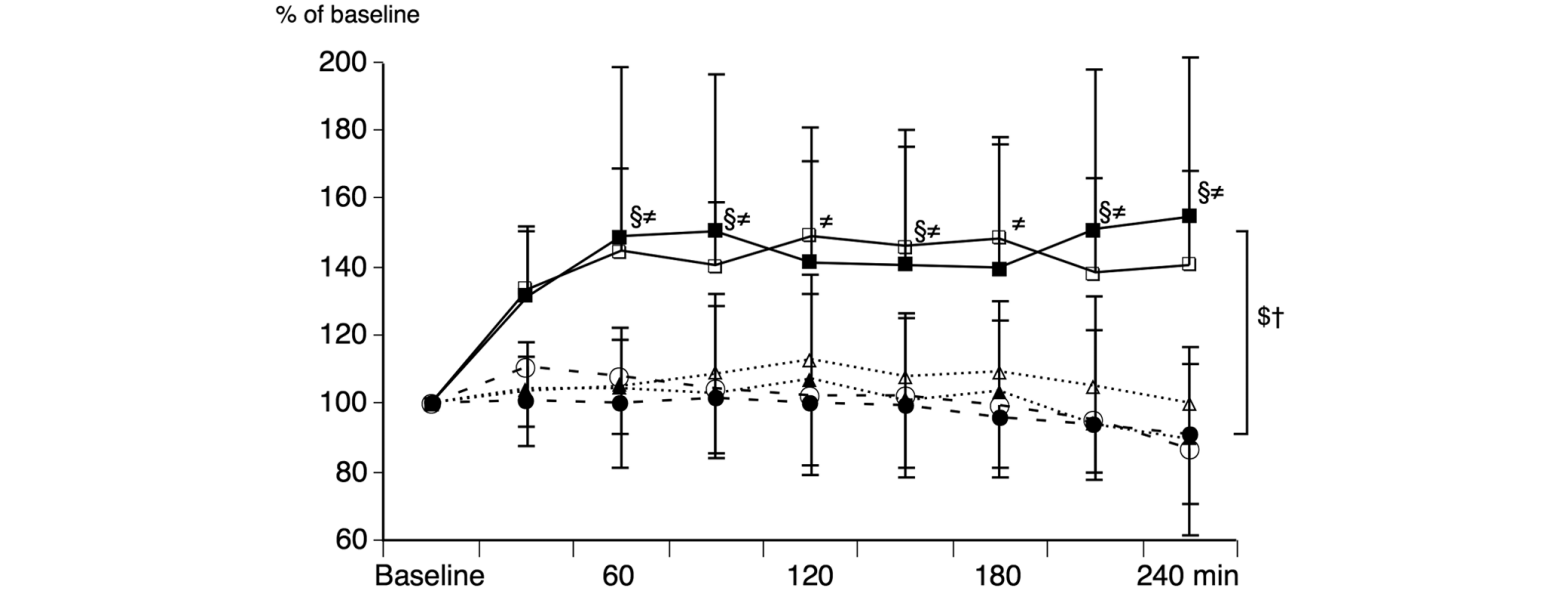
Relative changes in intestinal microcirculation and tissue oxygen tension (ptiO_2_). Black squares indicate relative changes in microcirculatory blood flow (MBF) in the goal-directed colloid fluid therapy (GD-C) group. Open squares indicate relative changes in ptiO_2 _in the GD-C group. Black triangles indicate relative changes in MBF in the goal-directed Ringer lactate fluid therapy (GD-RL) group. Open triangles indicate relative changes in ptiO_2 _in the GD-RL group. Black circles indicate relative changes in MBF in the restricted Ringer lactate fluid therapy (R-RL) group. Open circles indicate relative changes in ptiO_2 _in the R-RL group. The R-RL group received 3 mL/kg per hour of lactated Ringer solution throughout the entire experiment. The GD-RL group received 3 mL/kg per hour of lactated Ringer solution plus 250 mL of lactated Ringer solution if mixed venous oxygen saturation (SvO_2_) was less than 60%. The GD-C group received 3 mL/kg per hour of lactated Ringer solution plus 250 mL of hydroxyethyl starch (130/0.4) if SvO_2 _was less than 60%. Baseline was set at 100% for all parameters. Significant differences (*P *< 0.05) for area under the curve: ^†^R-RL versus GD-C, ^$^GD-RL versus GD-C. Significant differences (*P *< 0.05) for analysis of variance for repeated measurements (Tukey *post hoc *test): ^≠^R-RL versus GD-C, ^§^GD-RL versus GD-C.

Jejunum tissue oxygen tension (Figures 3 and 4) decreased by 15% in the R-RL group but remained unchanged in the GD-RL group. In the GD-C group, it increased by more than 40%, virtually in parallel with mucosal microcirculatory flow, and remained high until the end. Jejunal mucosa carbon dioxide tension (Figure 3) remained almost unchanged in the two crystalloid fluid groups but decreased by 10% in the colloid group.

**Figure 5:**
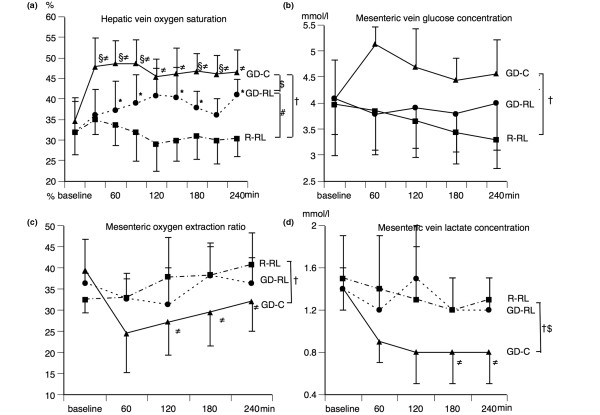
Splanchnic oxygenation parameters. **(a) **Changes in hepatic vein oxygen saturation (mean ± SD) before (baseline) and during the different fluid treatment strategies. **(b) **Changes in mesenteric vein glucose (mean ± SD) before (baseline) and during the different fluid treatment strategies. **(c) **Changes in mesenteric oxygen extraction ratio (mean ± SD) before (baseline) and during the different fluid treatment strategies. **(d) **Changes in mesenteric vein lactate (mean ± SD) before (baseline) and during the different fluid treatment strategies. The restricted Ringer lactate fluid therapy (R-RL) group received 3 mL/kg per hour of lactated Ringer solution throughout the entire experiment. The goal-directed Ringer lactate fluid therapy (GD-RL) group received 3 mL/kg per hour of lactated Ringer solution plus 250 mL of lactated Ringer solution if mixed venous oxygen saturation (SvO_2_) was less than 60%. The goal-directed colloid fluid therapy (GD-C) group received 3 mL/kg per hour of lactated Ringer solution plus 250 mL of hydroxyethyl starch (130/0.4) if SvO_2 _was less than 60%. Significant differences (*P *< 0.05) for area under the curve: ^#^R-RL versus GD-RL, ^†^R-RL versus GD-C, ^$^GD-RL versus GD-C. Significant differences (*P *< 0.05) for analysis of variance for repeated measurements (Tukey *post hoc *test): *R-RL versus GD-RL, ^≠^R-RL versus GD-C, ^§^GD-RL versus GD-C. SD, standard deviation.

Systemic oxygen delivery increased by almost 40% in the GD-C group and 20% in the GD-RL group, and systemic oxygen extraction ratio decreased by 25% in the GD-C group and 15% in the GD-RL group. Both parameters decreased in the R-RL group (Table 2). Hepatic venous oxygen saturation (Figure 5) increased rapidly by 40% in the GD-C group but increased slowly in the GD-RL group and decreased in the R-RL group. Mesenteric oxygen extraction ratio (Figure 5) decreased by more than 20% in the GD-C group but increased by 10% in the two crystalloid fluid groups. Lactate levels in the mesenteric vein (Figure 5) remained unchanged in the R-RL and GD-RL groups and decreased by 50% in the GD-C group. Hepatic vein lactate was similar in all groups. Glucose concentration in the mesenteric vein decreased by 15% in the R-RL group, was virtually unchanged in the GD-RL group, and increased by 12% in the GD-C group. Arterial Hb (Table 2) increased slightly in the R-RL group but decreased by approximately 10% in the two goal-directed groups.

**Table 2:** Oxygen delivery, extraction, and other variables

	sDO_2_I^a, b ^(mL/kg per minute)	sER^a-c ^(percentage)	mDO_2_I^b ^(mm Hg/kg per minute)	Mesent lac (mmol/L)	Arterial Hb^b ^(g/L)	Arterial pH	Arterial pO_2 _(mm Hg)	Arterial pCO_2 _(mm Hg)	Arterial BE (mmol/L)
Restricted Ringer lactate solution (R-RL)									
0 minutes	109 ± 11	49 ± 5	25 ± 2	1.5 ± 0.4	101 ± 7	7.51 ± 0.02	138 ± 9	36 ± 2	5.4 ± 0.9
60 minutes	103 ± 11	48 ± 6	26 ± 2	1.4 ± 0.6	102 ± 6	7.51 ± 0.02	135 ± 10	36 ± 3	5.3 ± 1.2
180 minutes	97 ± 12	50 ± 6	21 ± 2	1.2 ± 0.3	105 ± 9	7.5 ± 0.02	134 ± 15	36 ± 2	4.5 ± 1.7
240 minutes	97 ± 12	50 ± 4	20 ± 1	1.3 ± 0.2	104 ± 6	7.49 ± 0.04	135 ± 12	37 ± 2	4.3 ± 2.0

Goal-directed Ringer lactate solution (GD-RL)									
0 minutes	109 ± 17	51 ± 4	26 ± 6	1.4 ± 0.2	100 ± 10	7.52 ± 0.05	132 ± 11	37 ± 3	6.3 ± 1.9
60 minutes	116 ± 19	50 ± 4	30 ± 8	1.2 ± 0.2	99 ± 10	7.52 ± 0.04	132 ± 10	36 ± 2	6.4 ± 2.0
180 minutes	134 ± 26	44 ± 4	27 ± 6	1.2 ± 0.3	96 ± 6	7.49 ± 0.037	127 ± 10	38 ± 2	5.6 ± 2.0
240 minutes	130 ± 18	42 ± 7	26 ± 7	1.2 ± 0.3	90 ± 8	49 ± 0.03	126 ± 9	38 ± 3	5.1 ± 2.1

Goal-directed colloid solution (GD-C)									
0 minutes	116 ± 18	50 ± 6	26 ± 6	1.4 ± 0.4	97 ± 12	7.52 ± 0.03	135 ± 12	37 ± 3	5.8 ± 1.2
60 minutes	141 ± 22^c^	38 ± 5^b, c^	35 ± 8	0.9 ± 0.2	86 ± 10	7.5 ± 0.02	132 ± 17	38 ± 3	5.7 ± 1.4
180 minutes	151 ± 33^d^	38 ± 4^d, e^	29 ± 6^d^	0.8 ± 0.3	89 ± 11^d^	7.49 ± 0.02	131 ± 14	38 ± 1	5.0 ± 1.3
240 minutes	158 ± 38^d^	37 ± 5^d^	27 ± 5^d^	0.8 ± 0.3	87 ± 12^d^	7.49 ± 0.02	128 ± 16	37 ± 1	4.6 ± 1.0

Data are presented as mean ± standard deviation. t = 0 baseline values are from before the start of the respective fluid therapy. The measurements were performed hourly for 4 hours. Significant differences (*P *< 0.05) for area under the curve: ^a^R-RL versus GD-RL, ^b^R-RL versus GD-C, ^c^GD-RL versus GD-C. Significant differences (*P *< 0.05) for analysis of variance for repeated measurements (Tukey *post hoc *test): ^d^R-RL versus GD-C, ^e^GD-RL versus GD-C. The R-RL group received 3 mL/kg per hour of lactated Ringer solution throughout the entire experiment. The GD-RL group received 3 mL/kg per hour of lactated Ringer solution plus 250 mL of lactated Ringer solution if SvO_2 _was less than 60%. The GD-C group received 3 mL/kg per hour of lactated Ringer solution plus 250 mL of hydroxyethyl starch (130/0.4) if SvO_2 _was less than 60%. BE, standard base excess; Hb, hemoglobin concentration; mDO_2_I, mesenteric oxygen delivery; mesent lac, mesenteric venous lactate; pCO_2_, carbon dioxide partial pressure; pO_2_, oxygen partial pressure; sDO_2_, systemic oxygen delivery index; sER, systemic oxygen extraction ratio.

## Discussion

In this study, the effects of three different fluid regimens on systemic and regional blood flow as well as intestinal microcirculation and tissue oxygen tension were investigated during major abdominal surgery in pigs. The two groups receiving goal-directed fluid therapy (the GD-RL and GD-C groups) had increased cardiac output and increased regional blood flow to the SMA compared with the group receiving a restricted fluid regimen (the R-RL group). However, the effects of the two goal-directed fluid regimens were remarkably different in regard to microcirculatory blood flow, tissue oxygen tension, and metabolic markers in the small bowel. The first bolus of goal-directed administration of colloids resulted in a 30% increase in microcirculatory blood flow in the small bowel mucosa with a concomitant increase in tissue oxygen tension (30%), an increase in mesenteric vein glucose (12%), and decreases in mesenteric lactate (50%), mesenteric oxygen extraction (20%), and intestinal carbon dioxide (Figures 3, 4 and 5). On the other hand, even repeated boluses of RL in the GD-RL group did not increase microcirculatory blood flow in the small bowel mucosa and showed virtually no effect on tissue oxygenation, intestinal carbon dioxide, mesenteric lactate, or glucose levels. Comparable PPV, SV, and Hb values at the end of the study suggest similarly appropriate intravascular fluid volume in the two GDT groups.

Although systemic and regional blood flow increased significantly over time in the GD-RL group, the goal of SvO_2 _of at least 60% was not achieved in this group. It could be argued that if even larger amounts of crystalloids (more than 15 mL/kg per hour) had been administered microcirculatory blood flow in the small bowel might have increased comparably to the colloid group. However, dynamic systemic hemodynamic parameters such as PPV, SV, and Hb suggest that the two goal-directed groups had similar intravascular fluid volume at the end of the study. Furthermore, despite increasing systemic and regional blood flow over time, no trend of improvement in intestinal tissue oxygen tension or microcirculatory blood flow (Figure 3) in the goal-directed crystalloid group was found. In addition, even larger amounts of crystalloids (over 20 mL/kg per hour) did not increase perioperative small intestinal tissue oxygen tension [25].

Intestinal autoregulation does not explain the differences between the groups and suggests that the different pharmacological properties of the two fluid types, lactated Ringer solution and 6% HES (130/0.4), were to a large extent responsible for the effects on the intestinal microcirculation. RL is distributed within the whole extracellular space (that is, three fourths of the administered amount leave the intravascular space within minutes [26], thus expanding the extravascular space with interstitial fluid accumulation instead of increasing nutritive microcirculatory perfusion). Colloids, on the other hand, increase the intravascular volume as long as the endothelial glycocalix is competent [26] and thus may result in increased microcirculatory perfusion. The results are also in accordance with studies from Lang and colleagues [27] and Mythen and colleagues [5]. Lang and colleagues showed that colloid administration resulted in increased skeletal muscle oxygen tension in patients but that RL did not. Mythen and colleagues measured gastrointestinal blood flow indirectly by gastric tonometry in patients undergoing cardiac surgery. The authors found improved gastric mucosa pH and outcome in patients receiving goal-directed administration of colloids compared with control patients [5]. In addition, several other clinical studies have reported improved outcome after major surgery in patients receiving goal-directed HES [8,28-31] compared with conventional fluid therapy. However, none of these studies measured microcirculatory blood flow, tissue oxygen tension, or regional metabolic parameters directly in the gastrointestinal tract.

The strength of the present study is the combination of various methods to explore small intestinal microcirculation, oxygen transport, and markers of oxygen metabolism simultaneously. Interestingly, mesenteric vein glucose decreased in the fluid-restricted animals but increased in the colloid group. This is in accordance with a previous study from Krejci and colleagues [32], which showed that a reduction in intestinal glucose levels was an early sign (earlier than increased lactate) of gastrointestinal hypoperfusion.

In light of the microcirculatory effects of colloid administration in the present study, intestinal tissue oxygen pressure as well as mesenteric metabolic markers indicate augmented oxygen supply and sufficient cellular substrate. These findings may explain the basic, tissue-level mechanisms by which goal-directed administration of colloids has a beneficial impact on outcome.

The lack of effect of any of the fluid regimens used in this study on blood flow in the celiac trunk and the hepatic artery compared with a marked increase in systemic and SMA blood flows was an unanticipated finding and demonstrates once again the heterogeneous distribution of blood flow during different insults [13,20,33]. This underlines the fact that it is not appropriate to assume that changes in systemic, regional, and microcirculatory blood flow occur in unison under nonseptic conditions.

The main limitation of this study is the relatively short observation time (4 hours), which is too short to verify the effect of the respective fluid regimens on outcome. However, the aim of this study was to identify possible mechanisms and compare the *acute *effects of restricted and goal-directed fluid therapy on microcirculatory blood flow as well as several markers of tissue oxygenation and metabolism in the gut.

The study was performed in an animal model because direct measurements of regional and local microcirculatory blood flow in patients are invasive, time-consuming, and require special skills and instruments that are not readily available at the bedside. This is also the reason why no clinical study, to our knowledge, has measured the direct effects of goal-directed fluid therapy with crystalloid and colloid fluids on intestinal microcirculation, tissue oxygen tension, and metabolism. Therefore, the pathophysiologic background of improved outcome with goal-directed fluid therapy based on colloids was so far largely unknown. We chose the pig for this study because of its anatomical and physiologic similarity to humans with respect to the cardiovascular system and the digestive tract [34].

Another limitation of this experimental study concerns the choice of treatment target for fluid therapy. We do not suggest that the target of mixed venous saturation above 60%, as used in this study, is valid for patients undergoing major surgery. First, the target of 60% for mixed venous saturation seems rather low in patients, but it is ambitious in pigs because normal SvO_2 _in pigs is lower than in humans [34]. Second, mixed venous saturation measurements require a pulmonary artery catheter, which appears invasive for patients undergoing uncomplicated major surgery, particularly since other target parameters have been evaluated [8-10]. Last but not least, based on the currently available data, SV optimization for intravenous fluid challenges is the best evaluated method for individualized goal-directed fluid therapy and therefore seems preferable for human studies. However, for the purpose of this study, we considered this method very reliable and our animals were all instrumented with pulmonary artery catheters with continuous SvO_2 _monitoring.

An additional limitation of the study is that the two goal-directed groups received the same size of fluid bolus with identical lockout times. Thus, the GD-RL group may have needed more time to receive a hemodynamically equally effective amount of fluid. The aim of goal-directed fluid therapy (GDT) in the present study was to achieve a physiologic goal over a certain time period. The size of the intravenous fluid bolus administered in this study corresponds to 580 mL in a 70-kg patient and reflects clinical practice at our institution in hemodynamically stable patients (bolus infusion of approximately 500 mL). After such a bolus, a re-evaluation of the hemodynamics of the patient is mandatory. Thus, also because fluid distribution after a rapid bolus administration needed some time, it was not considered advisable to have a lockout time shorter than 30 minutes. A slight delay in achieving the goal parameter in the GD-RL group was found to be acceptable under the circumstances, particularly since it has been shown in a previous study that early aggressive fluid administration during surgery with crystalloids (> 20 mL/kg per hour) did not improve intestinal tissue oxygen tension compared with fluid restriction [25].

The present study indicates that, in the small bowel, fluid restriction as reflected by the data from the R-RL group results in impaired microcirculation and decreased tissue oxygen tension and cellular oxygen metabolism. Goal-directed fluid therapy with crystalloids had virtually no beneficial effects on either the intestinal microcirculation or the tissue oxygen tension but requires considerable amounts of fluids. However, excessive fluid administration may result in interstitial fluid accumulation and weight gain. Significant perioperative weight gain, however, results in increased mortality [35].

## Conclusions

The results from this animal study directly show for the first time that goal-directed fluid therapy with colloids increases intestinal microcirculatory blood flow and tissue oxygen tension compared with GDT with lactated Ringer solution or fluid restriction. Neither goal-directed crystalloid treatment nor fluid restriction had beneficial effects on intestinal microcirculation or tissue oxygen tension. In addition, mesenteric venous glucose and lactate concentrations suggest that intestinal cellular substrate levels were increased in the colloid group compared with the other groups. Consequently, the presented data support the notion that perioperative goal-directed fluid therapy with colloids might be beneficial to restore intravascular volume depletion, intestinal microcirculatory blood flow, and tissue oxygen delivery during major abdominal surgery.

## Key messages

• Colloids (hydroxyethyl starch 130/0.4) markedly increased microcirculatory blood flow and tissue oxygen tension in the small intestinal mucosa.

• Colloids decreased intestinal carbon dioxide gap, decreased mesenteric venous lactate, and increased mesenteric venous glucose concentration, suggesting improved intestinal cellular substrate levels.

• Colloids significantly increased mixed venous saturation with less fluid administered compared with crystalloids.

• Different fluid therapy regimens had no apparent effects on hepatic arterial blood flow, indicating sufficient liver tissue oxygenation even during restricted fluid administration.

• The results of this animal study suggest possible mechanisms for improved outcome after goal-directed therapy with colloids in major abdominal surgery in patients. This hypothesis, however, requires further studies.

## Abbreviations

ANOVA: analysis of variance; CaO_2_: arterial oxygen content; CI: cardiac index; CVP: central venous pressure; GD-C: goal-directed colloid fluid therapy; GD-RL: goal-directed Ringer lactate fluid therapy; GDT: goal-directed fluid therapy; Hb: hemoglobin concentration; HES: hydroxyethyl starch; HVP: hepatic vein pressure; LDF: laser Doppler flowmetry; MAP: mean arterial blood pressure; PAP: pulmonary artery pressure; PCWP: pulmonary capillary wedge pressure; pO_2_: oxygen partial pressure; PPV: pulse pressure variation; RL: Ringer lactate; R-RL: restricted Ringer lactate fluid therapy; SMA: superior mesenteric artery; SMAI: superior mesenteric artery flow index; SO_2_: arterial oxygen saturation; SV: stroke volume; SvO_2_: mixed venous oxygen saturation; SVRI: systemic vascular resistance index.

## Competing interests

The authors declare that they have no competing interests.

## Authors' contributions

LBH participated in experimental design, animal preparation, performance and supervision of experimental work, preliminary analysis of the data, and writing of the manuscript and provided supervision and oversight of the entire project. OK participated in experimental design, animal preparation, performance and supervision of experimental work, and analysis of the data and helped to draft the manuscript. MA participated in animal preparation, performance and supervision of experimental work, and preliminary analysis of the data and helped to draft the manuscript. SB participated in animal preparation, performance and supervision of experimental work, and preliminary analysis of the data. AK consulted on the experimental design, assisted with statistics, and participated in drafting the manuscript. GHS provided assistance with and consulted on the experimental design, made a substantial contribution to the manuscript (in particular, the Discussion section), and served as senior advisor. All authors read and approved the final manuscript.

## References

[B1] Bozzetti F, Gianotti L, Braga M, Di Carlo V, Mariani L (2007). Postoperative complications in gastrointestinal cancer patients: the joint role of the nutritional status and the nutritional support. Clin Nutr.

[B2] Law WL, Choi HK, Lee YM, Ho JW, Seto CL (2007). Anastomotic leakage is associated with poor long-term outcome in patients after curative colorectal resection for malignancy. J Gastrointest Surg.

[B3] Mythen MG, Webb AR (1994). Intra-operative gut mucosal hypoperfusion is associated with increased post-operative complications and cost. Intensive Care Med.

[B4] Bland RD, Shoemaker WC (1985). Probability of survival as a prognostic and severity of illness score in critically ill surgical patients. Crit Care Med.

[B5] Mythen MG, Webb AR (1995). Perioperative plasma volume expansion reduces the incidence of gut mucosal hypoperfusion during cardiac surgery. Arch Surg.

[B6] Theodoropoulos G, Lloyd LR, Cousins G, Pieper D (2001). Intraoperative and early postoperative gastric intramucosal pH predicts morbidity and mortality after major abdominal surgery. Am Surg.

[B7] Ivatury RR, Simon RJ, Islam S, Fueg A, Rohman M, Stahl WM (1996). A prospective randomized study of end points of resuscitation after major trauma: global oxygen transport indices versus organ-specific gastric mucosal pH. J Am Coll Surg.

[B8] Gan TJ, Soppitt A, Maroof M, el-Moalem H, Robertson KM, Moretti E, Dwane P, Glass PS (2002). Goal-directed intraoperative fluid administration reduces length of hospital stay after major surgery. Anesthesiology.

[B9] Pearse R, Dawson D, Fawcett J, Rhodes A, Grounds RM, Bennett ED (2005). Early goal-directed therapy after major surgery reduces complications and duration of hospital stay. A randomised, controlled trial [ISRCTN38797445]. Crit Care.

[B10] Lopes MR, Oliveira MA, Pereira VO, Lemos IP, Auler JO Jr, Michard F (2007). Goal-directed fluid management based on pulse pressure variation monitoring during high-risk surgery: a pilot randomized controlled trial. Crit Care.

[B11] Sinclair S, James S, Singer M (1997). Intraoperative intravascular volume optimisation and length of hospital stay after repair of proximal femoral fracture: randomised controlled trial. BMJ.

[B12] Wakeling HG, McFall MR, Jenkins CS, Woods WG, Miles WF, Barclay GR, Fleming SC (2005). Intraoperative oesophageal Doppler guided fluid management shortens postoperative hospital stay after major bowel surgery. Br J Anaesth.

[B13] Ali SZ, Bracht H, Krejci V, Beck M, Stalder M, Hiltebrand L, Takala J, Brandt S, Jakob SM (2008). The immediate and sustained effects of volume challenge on regional blood flows in pigs. Anesth Analg.

[B14] Grocott MP, Mythen MG, Gan TJ (2005). Perioperative fluid management and clinical outcomes in adults. Anesth Analg.

[B15] Fleischmann E, Herbst F, Kugener A, Kabon B, Niedermayr M, Sessler DI, Kurz A (2006). Mild hypercapnia increases subcutaneous and colonic oxygen tension in patients given 80% inspired oxygen during abdominal surgery. Anesthesiology.

[B16] Ratnaraj J, Kabon B, Talcott MR, Sessler DI, Kurz A (2004). Supplemental oxygen and carbon dioxide each increase subcutaneous and intestinal intramural oxygenation. Anesth Analg.

[B17] Kiel JW, Riedel GL, DiResta GR, Shepherd AP (1985). Gastric mucosal blood flow measured by laser-Doppler velocimetry. Am J Physiol.

[B18] Shepherd AP, Riedel GL (1982). Continuous measurement of intestinal mucosal blood flow by laser-Doppler velocimetry. Am J Physiol.

[B19] Krejci V, Hiltebrand LB, Sigurdsson GH (2006). Effects of epinephrine, norepinephrine, and phenylephrine on microcirculatory blood flow in the gastrointestinal tract in sepsis. Crit Care Med.

[B20] Hiltebrand LB, Krejci V, Banic A, Erni D, Wheatley AM, Sigurdsson GH (2000). Dynamic study of the distribution of microcirculatory blood flow in multiple splanchnic organs in septic shock. Crit Care Med.

[B21] Banic A, Krejci V, Erni D, Wheatley AM, Sigurdsson GH (1999). Effects of sodium nitroprusside and phenylephrine on blood flow in free musculocutaneous flaps during general anesthesia. Anesthesiology.

[B22] Hiltebrand LB, Krejci V, tenHoevel ME, Banic A, Sigurdsson GH (2003). Redistribution of microcirculatory blood flow within the intestinal wall during sepsis and general anesthesia. Anesthesiology.

[B23] Hiltebrand LB, Krejci V, Jakob SM, Takala J, Sigurdsson GH (2007). Effects of vasopressin on microcirculatory blood flow in the gastrointestinal tract in anesthetized pigs in septic shock. Anesthesiology.

[B24] Kimberger O, Arnberger M, Brandt S, Plock J, Sigurdsson GH, Kurz A, Hiltebrand LB (2009). Goal – directed colloid administration improves the microcirculation of healthy and perianastomotic colon. Anesthesiology.

[B25] Hiltebrand LB, Pestel G, Hager H, Ratnaraj J, Sigurdsson GH, Kurz A (2007). Perioperative fluid management: comparison of high, medium and low fluid volume on tissue oxygen pressure in the small bowel and colon. Eur J Anaesthesiol.

[B26] Chappell D, Jacob M, Hofmann-Kiefer K, Conzen P, Rehm M (2008). A rational approach to perioperative fluid management. Anesthesiology.

[B27] Lang K, Boldt J, Suttner S, Haisch G (2001). Colloids versus crystalloids and tissue oxygen tension in patients undergoing major abdominal surgery. Anesth Analg.

[B28] Conway DH, Mayall R, Abdul-Latif MS, Gilligan S, Tackaberry C (2002). Randomised controlled trial investigating the influence of intravenous fluid titration using oesophageal Doppler monitoring during bowel surgery. Anaesthesia.

[B29] Venn R, Steele A, Richardson P, Poloniecki J, Grounds M, Newman P (2002). Randomized controlled trial to investigate influence of the fluid challenge on duration of hospital stay and perioperative morbidity in patients with hip fractures. Br J Anaesth.

[B30] Price J, Sear J, Venn R (2002). Perioperative fluid volume optimization following proximal femoral fracture. Cochrane Database Syst Rev.

[B31] Pearse R, Dawson D, Fawcett J, Rhodes A, Grounds RM, Bennett ED (2005). Changes in central venous saturation after major surgery, and association with outcome. Crit Care.

[B32] Krejci V, Hiltebrand L, Buchi C, Ali SZ, Contaldo C, Takala J, Sigurdsson GH, Jakob SM (2006). Decreasing gut wall glucose as an early marker of impaired intestinal perfusion. Crit Care Med.

[B33] Krejci V, Hiltebrand L, Banic A, Erni D, Wheatley AM, Sigurdsson GH (2000). Continuous measurements of microcirculatory blood flow in gastrointestinal organs during acute haemorrhage. Br J Anaesth.

[B34] Hannon JP, Bossone CA, Wade CE (1990). Normal physiological values for conscious pigs used in biomedical research. Lab Anim Sci.

[B35] Lowell JA, Schifferdecker C, Driscoll DF, Benotti PN, Bistrian BR (1990). Postoperative fluid overload: not a benign problem. Crit Care Med.

